# Lipid droplets as a novel cargo of tunnelling nanotubes in endothelial cells

**DOI:** 10.1038/srep11453

**Published:** 2015-06-22

**Authors:** Ksenia Astanina, Marcus Koch, Christian Jüngst, Andreas Zumbusch, Alexandra K. Kiemer

**Affiliations:** 1 Department of Pharmacy, Pharmaceutical Biology, Saarland University, Saarbrücken, Germany; 2 Leibniz Institute for New Materials, Saarbrücken, Germany; 3 Department of Chemistry, Konstanz University, Konstanz, Germany

## Abstract

Intercellular communication is a fundamental process in the development and functioning of multicellular organisms. Recently, an essentially new type of intercellular communication, based on thin membrane channels between cells, has been reported. These structures, termed intercellular or tunnelling nanotubes (TNTs), permit the direct exchange of various components or signals (e.g., ions, proteins, or organelles) between non-adjacent cells at distances over 100 μm. Our studies revealed the presence of tunnelling nanotubes in microvascular endothelial cells (HMEC-1). The TNTs were studied with live cell imaging, environmental scanning electron microscopy (ESEM), and coherent anti-Stokes Raman scattering spectroscopy (CARS). Tunneling nanotubes showed marked persistence: the TNTs could connect cells over long distances (up to 150 μm) for several hours. Several cellular organelles were present in TNTs, such as lysosomes and mitochondria. Moreover, we could identify lipid droplets as a novel type of cargo in the TNTs. Under angiogenic conditions (VEGF treatment) the number of lipid droplets increased significantly. Arachidonic acid application not only increased the number of lipid droplets but also tripled the extent of TNT formation. Taken together, our results provide the first demonstration of lipid droplets as a cargo of TNTs and thereby open a new field in intercellular communication research.

Intercellular communication is a fundamental process in the development and functioning of multicellular organisms. Known mechanisms of cell-to-cell interactions include exocytosis, microvesicles and the direct transfer of small cytoplasmic components via gap junctions^1^,^2^. However, these forms of intercellular communication are not always suitable. For example, endothelial cells, a cell type that forms the inner lining of blood vessels, cannot use simple exocytosis and diffusion to transfer signals because they must function under the constant flow of blood. Nevertheless, the coordinated reaction of endothelial cells to certain stimuli (growth factors, regulators of blood pressure, etc.) is crucial for angiogenesis (formation of new blood vessels), inflammation, and cardiovascular haemostasis^3^. Importantly, such coordinated functioning requires effective long-range transfer of signals.

Recently, an essentially new type of intercellular communication, based on thin membrane channels between mammalian cells, has been reported^4^. These structures, called intercellular or tunnelling nanotubes (TNTs), permit the direct exchange of various components or signals (e.g., ions, proteins and organelles) between non-adjacent cells at distances over 100 μm^5^. The first TNT-like structures, called cytonemes, were identified in 1999^6^. Until now, they were found in different types of cells, *in vitro* and *in vivo*, in tissues and even in intact solid tumours from patients^7^. TNTs have been proposed to function in the intercellular trafficking of organelles and signals over long distances. Thus, TNTs transfer lysosomes, mitochondria, proteins, calcium ions, and other signals^5^.

In the past decade, TNTs have been found to play a role in various diseases. In this context it has been shown that prions can be transferred between infected and naive neuronal cells through TNTs^8^. The TNT-like cytonemes of neutrophils have been shown to be capable of catching and holding pathogenic bacteria^9^. HIV infection of human macrophages leads to increased TNT formation. Presumably, the HIV particles then use the TNT network to spread between cells^10^. Moreover, it is thought that TNT formation can be stimulated not only by viruses but also by stress conditions (e.g., H_2_O_2_ or serum depletion), apoptosis, or inflammation^11^,^12^.

Elucidation of the role of TNTs in endothelial cell-to-cell communication started in 2010, when TNT-like structures were found between stressed human umbilical vein endothelial cells (HUVECs) and endothelial progenitor cells (EPCs)^13^. This connection was suggested to enable the intercellular transfer of mitochondria and lysosomes, which results in improved HUVEC viability both *in vitro* and *in vivo*^14^.

In the present study, we provide the first demonstration that tunnelling nanotubes in endothelial cells contain not only lysosomes and mitochondria but also lipid droplets.

## Results

### Tunnelling nanotubes connect endothelial cells

**Figure 1:**
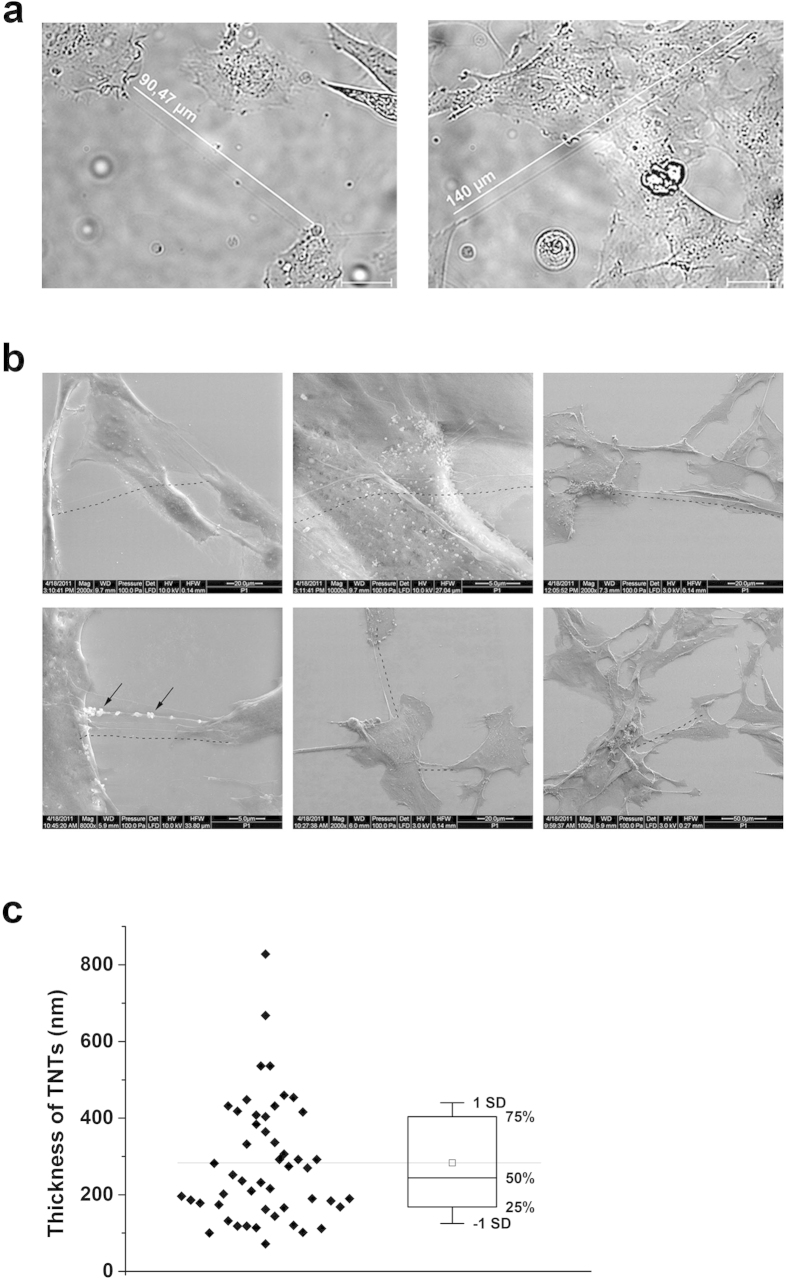
TNT-like structures connect endothelial cells (HMEC-1). (**a**) Brightfield images of TNTs in HMEC-1. TNTs connect cells at distances over 100 μm. White lines are placed parallel to TNT-like structures and illustrate the length of the corresponding TNTs. Scale bars, 20 μm. (**b**) TNTs connecting HMEC-1 visualized by scanning electron microscopy (ESEM). Some TNTs clearly extend over other cells (upper panel), and some carry bubble-like structures (bottom panel, depicted by arrows). Dashed black lines are drawn parallel to TNT-like structures. (**c**) The average thickness of TNTs was estimated by high-resolution imaging of ten TNTs by ESEM. The thickness was measured at five positions at each TNT. The bottom and the top of the box plot represent the first and the third quartiles, the whiskers – standard deviation (SD).

**Figure 2:**
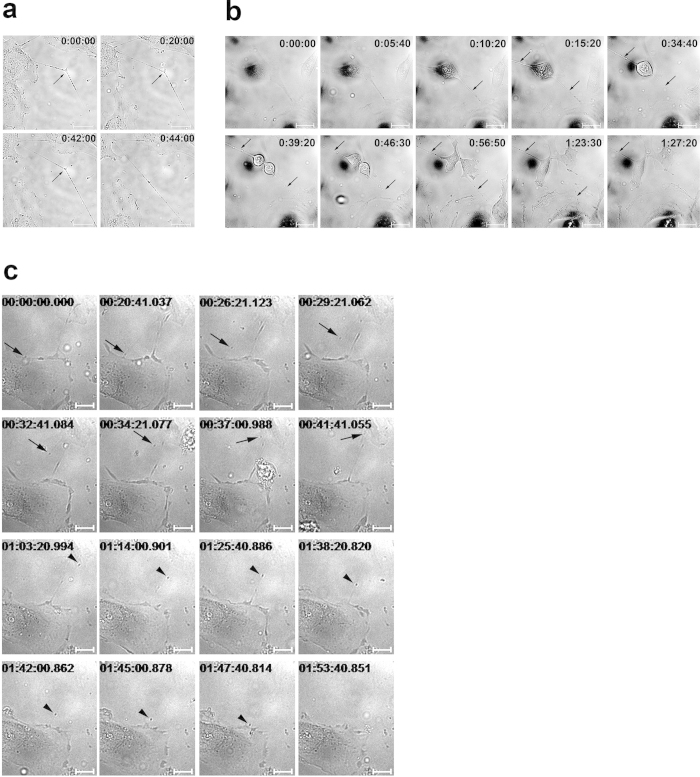
Still frames of TNT live imaging. (**a**) TNTs between cells experience tension. Once the anchor point (located on the substrate or on another cell, depicted with arrows) in the middle of the TNT is detached, the TNT straightens. (**b**) TNTs are often formed and maintained during mitosis. The transport of optically dense granules, or nodules, is observed inside TNTs (TNTs are depicted with arrows). (**c**) Transport of optically dense granules in TNTs between cells. Two examples of intercellular trafficking are shown (with arrow and arrowhead). Scale bars, 20 μm. Times are indicated as h:min:s (a, b) or h:min:s.ms (**c**). For video files, see the Supplementary Information.

**Figure 3:**
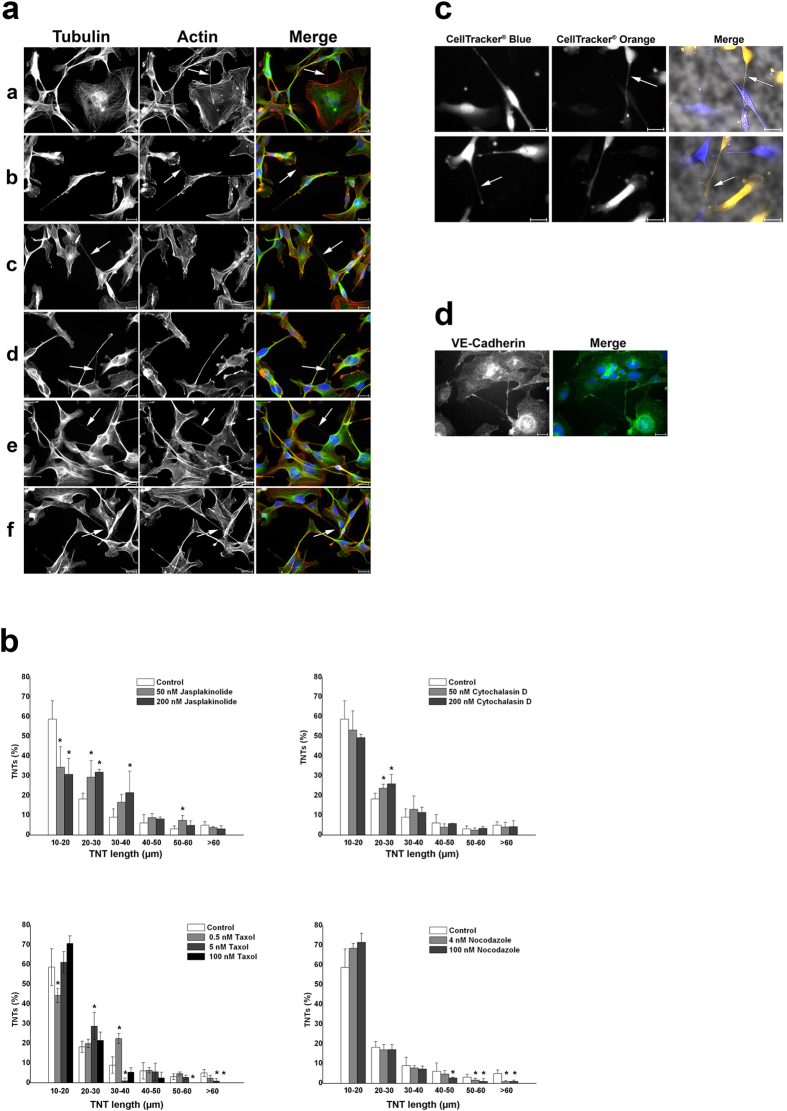
(**a**) Three types of TNTs were identified: actin-based (aa and ab), tubulin-based (ac and ad), and TNTs containing both types of cytoskeletal filaments (ae and af). HMEC-1 were grown on coverslips, fixed, and stained with an anti-tubulin antibody and rhodamine palloidin. Actin is shown in red; tubulin, in green. Merged images also show nuclear staining with DAPI. TNTs are depicted with arrows. (**b**) Treatment of HMEC-1 with cytoskeletal drugs causes changes in the distribution of TNT lengths. HMEC-1 were treated for 2 h with cytoskeletal drugs at the indicated concentrations, fixed, and then immunostained. The number and length of the TNTs were quantified in 40 random microscopic fields per treatment. The entire number of TNTs per treatment was set as 100%. The results from three independent experiments were used for statistical analysis. *p < 0.05. (**c**) Staining of HMEC-1 with two different CellTracker® dyes did not show any direct exchange of cytoplasm via TNTs. Merge images show additionally brightfield images. TNTs are depicted with arrows. (**d**) HMEC-1 were fixed and immunostained with an anti-VE-cadherin antibody (shown in green). Scale bars, 20 μm.

Human microvascular endothelial cells (HMEC-1) formed a variety of cell protrusions, including filopodia, lamellipodia, etc. HMEC-1 also formed thin membrane bridges between cells, which can be described as TNT-like structures (Fig. 1,2). The nanotubes differed in length (for length distribution, see Fig. 3b), the majority were between 10 and 30 μm, but some reached lengths of over 100 μm (Fig. 1a). Due to technical limitations, it is difficult to measure the thickness of these structures using optical microscopy; however, using ESEM imaging, the average thickness was estimated to be in the 180–400 nm range (Fig. 1c), which is in accordance with published data^12^. TNT-like structures observed in HMEC-1 also satisfied other TNT criteria^12^: they contained cytoskeletal filaments (see Fig. 3a) and were not attached to the substrate, which can be observed nicely in our live-cell recordings and scanning electron micrographs (Fig. 1b,2a, and Supplementary Information). Tunnelling nanotubes exhibited a remarkable stability and could be sustained for over one hour. ESEM imaging revealed the presence of bubble-like structures in the TNTs, which we thought might represent TNT cargo (e.g., organelles). Brightfield imaging showed that the TNTs carried cytoplasmic granules, or nodules, which were transported along the TNTs and between the cells (Fig. 2b,c). Interestingly, TNTs were often formed between the dividing cell and adjacent cells during cell mitosis (Fig. 2b and Supplementary Information). The nanotubes were stable during the whole mitotic process and were very long (over 100 μm). During mitosis, transport of the granules, or nodules, along the TNTs was observed (Fig. 2b and Supplementary Information).

### Cytoskeleton of tunnelling nanotubes in endothelial cells

To characterize the cytoskeleton of the TNTs, actin filaments and microtubules were stained in HMEC-1. We could detect three main types of TNTs in our cultures. The first type represented TNTs with only actin filaments inside; the second, with only tubulin; and the third, with both microtubules and actin filaments (Fig. 3a). Among the latter type, there was also diversity as the pattern of actin and tubulin along the TNTs differed among the nanotubes. The majority of the TNTs belonged to the third type, containing both microtubules and actin filaments. Nanotubes containing only actin were usually the shortest of the nanotubes.

To study the role of actin and tubulin in TNT formation and maintenance, we examined the effects of four cytoskeletal drugs. Two had filament-stabilizing properties (jasplakinolide for actin and taxol for microtubules), and two, filament-depolymerizing (cytochalasin D for actin and nocodazole for microtubules) (Fig. 3b). The applied concentrations of drugs were proved to be non-toxic (see Supplemetary Information). Because all of the compounds noticeably affected TNT length, these data indicate a clear involvement of cytoskeleton components in TNT formation and/or stability. Still, because the stabilizers and destabilizers sometimes elicited the same effect, a clear conclusion could not be drawn.

To clarify whether long TNTs “belong” to one of the connected cells or are formed by two cells equally, we stained two cell populations with two different colours of cytoplasmic marker (CellTracker®). The cell populations were then mixed and the images were taken 2 and 24 hours after seeding. At the first time point (2 hours after seeding) TNTs had formed, but there was no evidence for the direct exchange of cytoplasm between the cells: in the TNTs only one type of signal could be observed (Fig. 3c). After 24 hours all blue-labelled cells also had a weak orange signal. We suggest that this is due to diffusion of the dye, so no relevant conclusion can be drawn from the experiment at this later time point (Supplementary information).

Because VE-cadherin is one of the most prominent proteins responsible for cell adhesion and intercellular interactions, we stained HMEC-1 with an anti-VE-cadherin antibody^15^. The staining revealed intracellular localization of VE-cadherin, which is in accordance with published data for this and other VE-cadherin antibodies^15^,^16^. Remarkably, VE-cadherin accumulated along the TNTs.

### Lipid droplets are a novel cargo of tunnelling nanotubes

**Figure 4:**
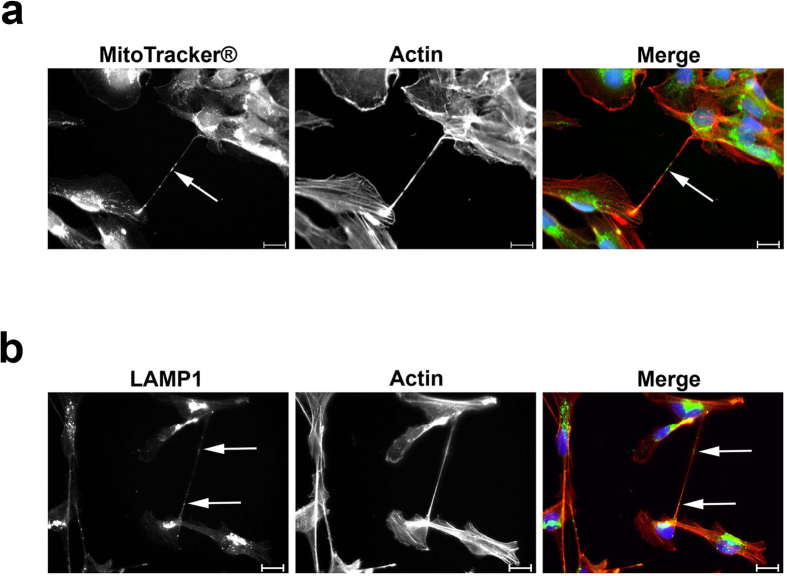
Lysosomes and mitochondria were identified as TNT cargo in HMEC-1. (**a**) HMEC-1 were stained with MitoTracker® and, subsequently, by immunofluorescence. Actin was stained with rhodamine phalloidin. Actin is shown in red; MitoTracker®, in green; and nuclei, in blue. (**b**) Lysosomes were stained by immunofluorescence with an anti-LAMP1 antibody. Actin was stained with rhodamine phalloidin. Actin is shown in red; LAMP1, in green; and nuclei, in blue. Scale bars, 20 μm.

**Figure 5:**
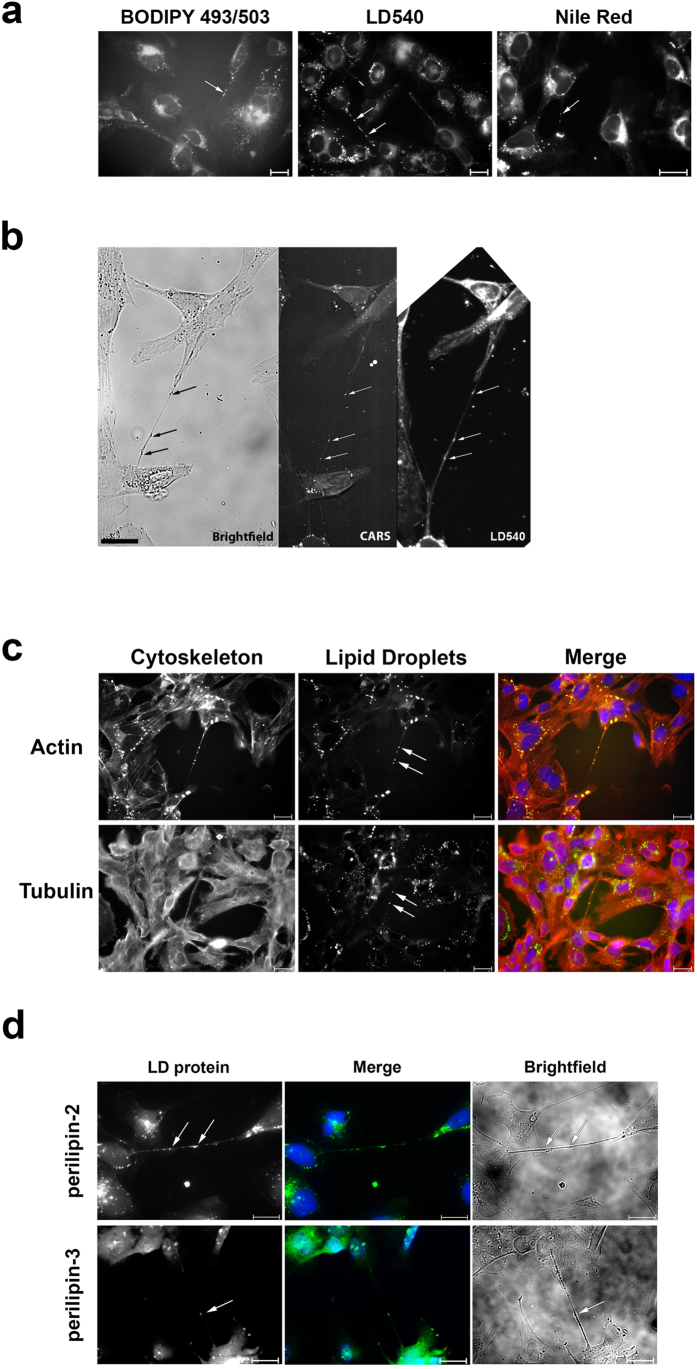
Lipid droplets (LD) were identified as a novel cargo of TNTs in HMEC-1. (**a**) Lipid droplets were stained with three different LD-specific dyes: BODIPY 493/503, LD540, and Nile red. LDs were present in TNT-like structures (shown with arrows). (**b**) The presence of LDs in TNTs was demonstrated with CARS. HMEC-1 were fixed and analysed by brightfield microscopy, then by CARS. Subsequently, the cells were taken from the microscope, washed, and stained with LD540. The same structures were observed in all three types of imaging (depicted with arrows). For overlays see Supplementary Information. (**c**) Lipid droplets were found in TNTs containing both actin and tubulin. Lipid droplets were stained with Nile red; tubulin, with an anti-tubulin antibody; and actin, with rhodamine phalloidin. Actin and tubulin are shown in red, LDs, in green. (**d**) LD-associated proteins (perilipin-3 and perilipin-2) were identified in TNTs. The proteins were stained by immunofluorescence with specific antisera. LDs are depicted with arrows. Scale bars, 20 μm.

Various cargoes have been observed in tunnelling nanotubes. Organelles (e.g., mitochondria and lysosomes^4^,^17^), proteins (e.g., prions^8^), viral particles (e.g., HIV-1^10^), and signals (e.g., calcium^18^) have been identified in intercellular nanotubular structures. We tested whether HMEC-1 TNTs carry mitochondria and lysosomes by staining the cells with MitoTracker® or an anti-LAMP1 antibody, and, in both cases, we could detect these organelles in the TNTs (Fig. 4). Interestingly, however, these organelles did not colocalize with the granules that were observed by brightfield imaging (Fig. 2). As a result, we wondered about the identity of these granules and finally identified them as lipid droplets (LDs) (Fig. 5). In fact, the majority of the optically dense granules observed in the tunnelling nanotubes were lipid droplets as shown by different experimental approaches.

We stained the lipid droplets with three different dyes (BODIPY 493/503, Nile red, and LD540), and all of the stains confirmed the presence of LDs in the TNTs (Fig. 5a). Additional coherent anti-Stokes Raman scattering spectroscopy (CARS) analysis strongly supported the idea that the observed granules were lipid droplets. CARS represents a novel way of visualizing lipid droplets inside cells without any label^19^. In fact, the granules, which were observed in brightfield microscopy, could be identified as lipid droplets using CARS, as well as by subsequent staining of the structures by LD540 (Fig. 5b, Supplementary Information). For further analyses, we chose Nile red as the main LD dye because the intensity of BODIPY 493/503 was rather weak, and staining with LD540 abolished lipid droplet movements in the cells. Lipid droplets were also present in the TNTs of primary endothelial cells (Supplementary Information).

We analysed the cytoskeleton of LD-carrying tunnelling nanotubes. We identified both actin and tubulin inside the nanotubes (Fig. 5c). To characterize the protein composition of the lipid droplets in the tunneling nanotubes, we stained for two prominent LD-associated proteins^20^, perilipin-2 and perilipin-3, and observed signals for both of them in the tunnelling nanotubes (Fig. 5d).

**Figure 6:**
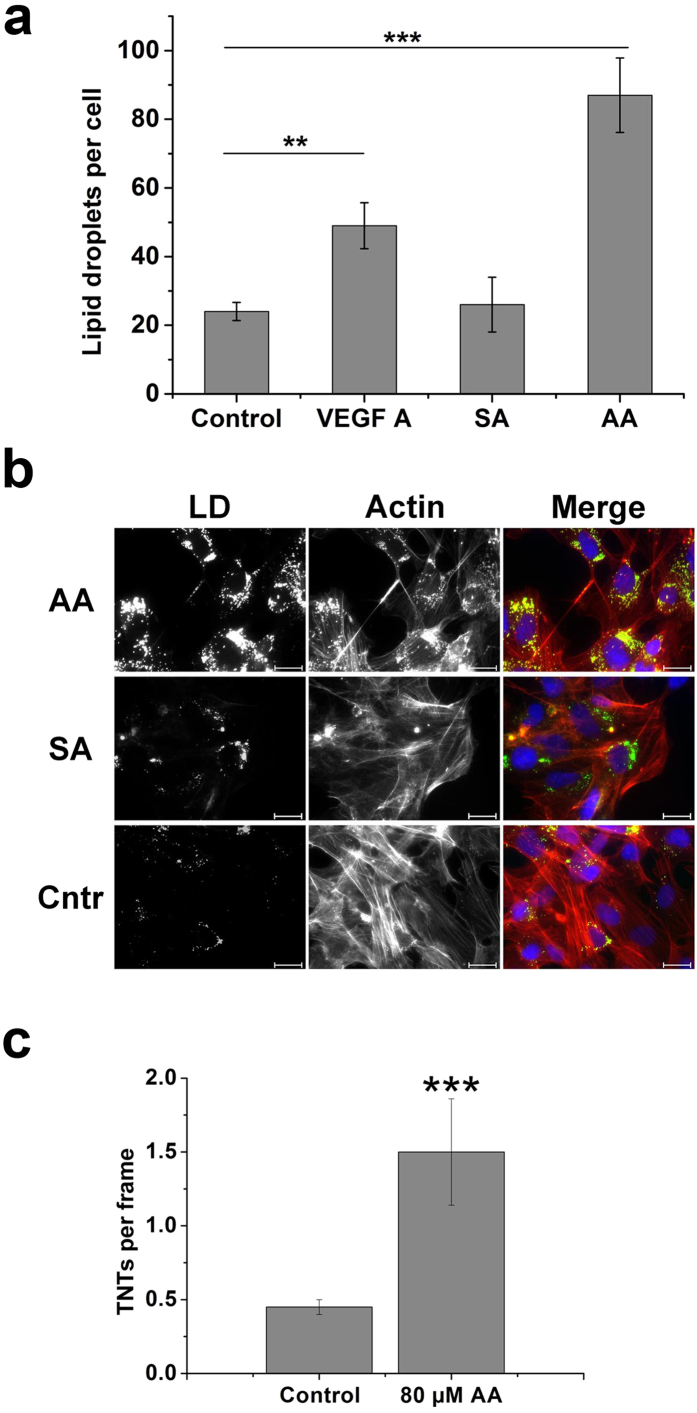
The number of LDs and TNTs increases in activated endothelial cells. (**a**) VEGF A and arachidonic acid (AA) lead to the increased number of LD in HMEC-1. HMEC-1 were treated with 100 ng/ml VEGF A or 80 μM AA or 80 μM SA for 24 h, fixed, and stained with Nile red. LDs were quantified with the ImageJ software. (**b**) Treatment of HMEC-1 with 80 μM arachidonic acid (AA) increases the number of LD in HMEC-1. The same concentration of stearic acid (SA) showed no effect. LD were stained with Nile red; actin, with rhodamine phalloidin. Actin is shown in red, LD – in green. Scale bars, 20 μm. (**c**) AA causes increased formation of TNTs in HMEC-1. After treatment with AA, HMEC-1 were fixed and cytoskeletal filaments were stained by immunofluorescence. The number of TNTs was determined in 20 random microscopic fields per treatment. (**a**, **c**) The data from three independent experiments were used for statistical analysis. Error bars denote s.e.m.. Significance: **p < 0.01, ***p < 0.001.

To study whether TNT formation and LD content changes in activated endothelial cells, we treated the cells with vascular endothelial growth factor (VEGF A). The treatment led to a significant increase in the number of lipid droplets in HMEC-1 (Fig. 6a). A similar but even stronger effect was achieved by treating HMEC-1 with arachidonic acid (Fig. 6a,b). The number of lipid droplets increased dramatically. The observed effect was specific because the application of stearic acid did not cause an increase in LD formation. Moreover, arachidonic acid increased not only lipid droplet formation but also TNT formation (Fig. 6c). This effect was also absent after treatment with stearic acid.

## Discussion

Tunnelling nanotubes are a recently described type of intercellular communication. Although they were first described 11 years ago, clear nomenclature and a comprehensive understanding of the function of these structures is still lacking. The primary characteristics of TNTs are thought to be their length (over 10 μm), the presence of cytoskeletal elements within the membranous connections, and the fact that they are not attached to the substrate^12^. However, the variety of TNT-like structures described to date suggests that this type of intercellular communication is rather heterogeneous. For example, there are both actin- and microtubule-based TNTs as well as open-ended and closed TNTs; some TNTs form from growing filopodia, whereas others result from cell dislodgement or retraction^5^. The question is whether all these diverse structures are similar enough to be classified as a single type of cellular connection termed tunnelling nanotubes.

Although some TNT-like structures exhibiting notable differences are combined into a single structural category, the fact that different names are often used for similar TNT-like connections is an additional problem. For example, TNT-like structures have been already described as cytonemes, intercellular nanotubes, membrane nanotubes, cellular bridges, streamers, and signalling filopodia^4^,^6^,^21^,^22^,^23^,^24^,^25^. Moreover, some TNTs strongly resemble retraction fibres and cannot be clearly distinguished from them^26^,^27^. A comprehensive study of TNTs in various cell types will allow the classification of TNT-like structures based on their specific functions, cargo, and morphological features.

In this study we analysed TNT-like structures in endothelial cells (HMEC-1). The observed structures meet the current criteria for TNTs: they connect cells over long distances (over 10 μm), are thin membranous channels, contain cytoskeletal filaments, and are not attached to the substrate^12^,^28^. We have shown that there are at least three types of TNTs: only actin-based, only microtubule-based, and containing both actin and tubulin. However, it is still unclear whether they truly represent three types of TNTs or instead represent three stages of TNT maturation. For example, it is plausible that TNTs first contain only actin fibres, with microtubules later growing inside and providing necessary stability and stiffness to the long TNTs. Notably, we have presented the first example of TNTs containing only microtubules; to date, only actin- or actin- and MT-based TNTs have been described^28^.

The most common approach to reveal whether a TNT belongs to a single cell or to both connected cells, is double-labelling with fluorescent dyes^29^,^30^. CellTracker® is a cell-permeable fluorescent dye, which is transformed in the cytoplasm into its cell-impermeable form. The dye stays retained in the cytoplasm for several cell cycles, which makes it a perfect tool for TNT studies. In a double-labelling experiment with CellTracker® we could see no direct exchange of cytoplasm between the cells. Still, we did occasionally see intracellular inclusions with “foreign” CellTracker® (Orange CellTracker® vesicles in Blue CellTracker®-loaded cells or vice versa). However, this does not prove the direct intercellular transfer of material because such exchange might also happen through exo- and endocytosis. At the same time, we cannot completely exclude the possibility of cytoplasm transfer via TNTs at later time points, possibly through more maturated nanotubes. Although we did not see the mixing of cytoplasm, this observation does not exclude the transfer of cargo (even organelles) through the TNTs because such transfer has already been described^4^. The mechanism of such transfer remains unclear. Tunnelling nanotubes, especially their sites of contact with the plasma membrane, are enriched in VE-cadherin, one of the main proteins comprising adherens junctions between endothelial cells^31^. Interestingly, although endothelial cells are highly motile during angiogenesis, VE-cadherin-mediated adherens junctions play a crucial role in angiogenesis^32^. Moreover, it has been demonstrated that the integrity of adherens junctions can be maintained during endothelial cell migration^33^. Thus, the formation of tunnelling nanotubes might be a mechanism to preserve adherens junctions and downstream signalling cascades between the cells during angiogenesis.

Notably, we observed that TNTs are often present during mitosis of cells. In this respect, they resemble retraction fibres, which have been previously described in *Potorous trydactylus* kidney (PtK2) cells^34^. Similar to retraction fibres, cytoplasmic granules or “nodules” were observed in the TNTs. It has been shown that such nodules in PtK2 cells are rich in actin, but a detailed structure has not yet been provided^26^. We have observed similar motile granules not only in retraction fibres of mitotic cells but also in many of the TNTs. The described granules were identified as lipid droplets. We demonstrated that the vast majority of the observed granules were lipid droplets using various techniques and can therefore argue that lipid droplets represent a new type of TNT cargo. By demonstrating the presence of lipid droplets in the TNTs of primary endothelial cells (HUVECs), we showed that this finding is not specific to immortalized endothelial cells. We have demonstrated that lipid droplets are actively moving along TNTs in both directions. Lipid droplets can be transported not only by motor proteins but also by cytoplasmic flow^35^. Taking into account the spatial confinement of the tunnelling nanotubes, it is possible that LDs are transported along TNTs by cytoplasmic streaming as well.

For a long time lipid droplets (also called lipid bodies) were believed to be simply lipid storage sites. In the last few years, lipid droplets have been recognized as a special type of organelle, with a complex biogenesis and structure and a variety of functions^36^. Thus, lipid droplets can serve as lipid reservoirs, centres for the synthesis of specific lipids, and as protein storage^37^. An increased number of lipid droplets is associated with pathological conditions such as inflammation, cancer, and hypoxia^38^. In endothelial cells, the biogenesis of lipid droplets increases under hypoxic conditions, which resemble the activation of angiogenesis in tumours^39^. The main role of lipid droplets in endothelial cells is presumably the synthesis of signalling lipids, such as eicosanoids (e.g., prostaglandins, leukotrienes and lipoxins). These mediators are synthesized from arachidonic acid and regulate various cellular functions, such as inflammation, metabolism, cell activation, migration, and apoptosis^40^. In endothelial cells, arachidonic acid regulates cell adhesion and migration, although it is not clear whether this regulation is facilitated through the eicosanoid signalling pathway^41^,^42^. In our study, arachidonic acid application resulted in an increased number of lipid droplets and TNT formation. The number of lipid droplets also increased upon treatment with vascular endothelial growth factor (VEGF). This effect could be either a result of an elevated *de novo* synthesis of lipid droplets or of the prolonged life time of LDs. Based on our data we cannot exclude any of these hypotheses but the first one seems more plausible, as it has been demonstrated in human monocytes that arachidonic acid induces the biogenesis of lipid droplets^43^.

Taken together, we propose that during endothelial cell activation (e.g., in angiogenesis), the number of lipid droplets increases, followed by the activation of certain signalling pathways (e.g., eicosanoid synthesis). This leads to increased cell motility as, for example, in angiogenesis. Migrating endothelial cells form additional tunnelling nanotubes to preserve adherens junctions and intercellular communication. Tunnelling nanotubes serve as highways for signals (e.g., lipid mediators) for coordinated cell migration. The presence of lipid droplets in TNTs may facilitate signalling because the synthesis of lipid mediators can occur “on site”, directly in the nanotube and close to the cell accepting the signal. Moreover, a direct intercellular transfer of lipid droplets might enhance such a signalling effect by transferring the whole machinery for eicosanoid synthesis.

Angiogenesis represents an interesting target for anti-tumour drugs. Thus, a detailed understanding of the mechanisms of angiogenic cell migration and communication might open new avenues for anti-cancer therapies. In general, intercellular communication via tunnelling nanotubes represents a novel and yet to be completely understood type of cell-to-cell interaction. Here, we describe a new type of TNT cargo, lipid droplets. The clarification of the role of lipid droplets in intercellular communication is a subject of further studies.

## Methods

### Antibodies, reagents, and constructs

The following antibodies were used: mouse anti-tubulin (Sigma), anti-LAMP1 (abcam), anti-perilipin-2 serum and anti-perilipin-3 serum (both from PROGEN Biotechnik), rabbit anti-VE-cadherin (New England BioLabs), and goat Alexa Fluor 488-conjugated anti-mouse and anti-rabbit (Life Technologies). For nuclei staining, DAPI (Sigma) was used; for actin filament staining, rhodamine phalloidin (Sigma). The following cytoskeletal drugs were applied: jasplakinolide (Sigma), cytochalasin D (Sigma), taxol (Cytoskeleton), and nocodazole (Sigma). For lipid droplet staining, two commercially available dyes were used: BODIPY 493/503 (Life Technologies) and Nile red (Sigma). CellTracker® and MitoTracker® were from Life Tecnologies; arachidonic acid from Sigma; and VEGF A, from R&D Systems.

### Synthesis of the fluorescent dye LD540

The synthesis of LD540 was conducted according to published procedures^44^,^45^. Briefly, 500 mg tetrahydroindole was dissolved in 3 ml dry dichloromethane and 1 ml of freshly distilled acetylchloride was added. The solution was heated to 40 °C for 1 h, before the solvent was removed and the residue dried. The intermediate was dissolved in 50 ml dichloromethane and 1.25 ml of triethylamine, and 1.2 ml of BF_3_-etherate was then added. After stirring for 16 h at room temperature, the solvent was removed and the resulting product cleaned using column chromatography. Consequently, 180 mg LD540 was obtained. The purity of LD540 was checked verified on basis of ^1^H-NMR spectroscopy and mass spectrometry and is estimated to be better than 95%.

### Cell culture

The human microvascular endothelial cell line (HMEC-1)^46^ was cultured in endothelial medium (Life Technologies) supplemented with 5% foetal calf serum, penicillin, streptomycin, and glutamine (Sigma). The cells were cultured at 37 °C in a humidified atmosphere containing 5% CO_2_. Primary human umbilical vein endothelial cells (HUVECs) were isolated from umbilical cords by digestion with 0.01% collagenase A solution (Roche) and grown in ECGM with supplement mix (Promocell) containing penicillin (100 U/ml), streptomycin (100 mg/ml), kanamycin (50 mg/ml), and 10% FCS (PAA). Umbilical veins were obtained with the consent of patients, and permission was given by the local ethics committee (#131/08). For experiments, cells were used at passage 3 or 4^47^,^48^.

### MTT cytotoxicity assay

MTT assay is a colorimetric test for cell viability allowing sensitive quantification of cytotoxicity^49^,^50^. The assay is based on the reduction of yellow tetrazole to purple formazan in living cells. HMEC-1 were seeded in a 96-well plate (50,000 cells/well). After 24 hours, the cells were treated with appropriate concentrations of test substances or with solvent only (the concentration of which corresponded to the highest concentration of DMSO or ethanol applied to drug-treated cells). Control cells remained untreated. After the treatment cells were rinsed and MTT solution was added (0.5 mg/ml). The plate was incubated at 37 °C for 2 hours in the absence of light. Thereafter, the solution was removed and 80 μl DMSO was added per well. Absorption was measured at 550 and 690 nm. The viability of the control cells was set as 100%.

### Immunofluorescence

Immunofluorescence was performed essentially as described previously^51^. HMEC-1 were grown on coverslips and fixed with 4% PFA. The cells were permeabilized with 0.1% Triton X-100 and blocked with 5% goat serum (Sigma). Primary and secondary antibodies were diluted in 5% goat serum and incubated with the cells in a wet chamber at room temperature. Coverslips were mounted with FluorSave™ (Merck Millipore). Images were obtained and analysed with an Axio Observer Z1 epifluorescence microscope (for details, see the Live cell imaging section).

### Double-labelling with CellTracker®

HMEC-1 were stained either with CellTracker® Blue, or CellTracker® Orange in accordance with the manufacturer’s protocol. Briefly, the dyes were diluted in medium up to final concentration 1 μM. The cells were incubated with the solution at 37 °C for 30 minutes. Thereafter, the dye was removed and the cells were rinsed. After trypsinization cells were mixed together (1:1 proportion) and plated on coverslips. The images were taken either 2 or 24 hours after seeding.

### Live cell imaging

For live cell imaging, HMEC-1 were grown in μ-Slides (VI^04^, ibidi). Thereafter, the cells were either analysed with bright field microscopy or pre-stained with the appropriate dye (CellTracker®, MitoTracker®, or lipid droplet dyes) in accordance with the manufacturer’s protocol. The cells were analysed with an Axio Observer Z1 epifluorescence microscope equipped with an AxioCam Mrm, Incubator XL multi-S1, TempModule S1, and CO2 Module S1 (all from Zeiss). All cell images were obtained using either a Fluar 40x/1.30 Oil M27 objective, or an LCI Plan-Neofluar 63x/1.30 Imm Korr Ph 3 M27 objective. All videos were recorded in a humidified atmosphere at 37 °C and 5% CO_2_. Data were obtained and analysed using the AxioVision software (Zeiss).

### Lipid droplet staining

For lipid droplet staining, the following dyes were used: BODIPY 493/503, Nile red, and LD540^45^,^52^,^53^. BODIPY 493/503 and LD540 were dissolved in ethanol; Nile red was dissolved in DMSO. Prior to staining, lipid droplet dyes were diluted in cell culture medium (to a final concentration of 1 μg/ml for BODIPY 493/503 and Nile red and 0.5 μg/ml for LD540). The cells were incubated with the dye at 37 °C for 10 minutes, rinsed, and either subjected to immunofluorescence staining or analysed directly with the fluorescence microscope (see the Live cell imaging section).

### Lipid droplet quantification

Lipid droplet quantification was performed similar to Mahammad & Parmryd^54^. HMEC-1 cells were cultured in μ-Slides (VI^04^, ibidi) and treated with AA, VEGF, SA or medium only (control cells). After 24 hours the cells were stained with Nile red and fixed. Images were done with an Axio Observer Z1 epifluorescence microscope (for details, see the Live cell imaging section). For each cell a Z-stack (0.3 μm step size) was aquired throughout the cell. The number of lipid droplets was analysed using the ImageJ software with the DropletFinder plugin. The LD size thresholds were set as 0.3 μm and 1.6 μm. For statistical analysis the data from three independent experiments were collected.

### CARS microscopy

CARS experiments were performed using a custom setup based on a Leica TCS SP5 multiphoton microscope equipped with a Leica 1.2 NA, 63x water immersion objective (Leica Microsystems, Germany). The data were recorded in transmission type geometry. CARS signals were collected by a Leica 0.55 NA air condenser and transmitted through suitable emission filters (680 SP + 641/75 BP, Semrock, United States). A Nd:YVO4 laser that delivering 7 ps pulses at a repetition rate of 76 MHz and a wavelength of 532 nm (HighQ Laser, Austria) was used to pump an optical parametric oscillator (APE, Germany), the signal beam of which was used as a the pump beam. The experiments were performed at the 2845 cm-1 resonance frequency of antisymmetric aliphatic CH_2_ stretching vibrations using the Stokes beam tuned to 817 nm. Residual 1064-nm light from the pump laser served as the Stokes beam. Excitation power at the laser exit was 520 mW for the pump beam and 230 mW for the Stokes beam.

### ESEM

HMEC-1 were grown on coverslips and fixed with fixation buffer (2% PFA, 0.05% glutaraldehyde in 0.01 M cacodylate buffer) for 30 minutes at RT. Thereafter, the samples were dehydrated in a graded ethanol series (30%, 50%, 70%, 80%, 90%, and 96% - for 10 minutes each, followed by 2 × 100% for 15 minutes), dried in hexamethyldisilazane (HMDS), and stored in an exicator. HMEC-1 cells were subsequently investigated by Environmental Scanning Electron Microscopy (ESEM) using an FEI Quanta 400 FEG (FEI Company, Hillsboro, USA) in low-vacuum mode at 100 Pa water vapour pressure. Secondary electron images were taken at 3 and 10 keV (spot size 3, final aperture size: 30 μm, dwell time: 30 μs per pixel, and image size: 1024 × 884 pixels).

To measure the thickness of TNTs, ten nanotubes were imaged with high resolution and the thickness of each was measured at five evenly distributed positions along the TNT. The measurement was made using ImageJ software.

### TNT quantification

After the different treatments, HMEC-1 were fixed and stained for actin filaments and microtubules. Thereafter, 40 images were taken at random positions on the microscopic slides for each treatment. All tunnelling nanotubes were manually measured in each image using AxioVision software. For cytoskeletal drugs analysis, the number of TNTs of a certain length was expressed as a percentage of all TNTs. For arachidonic acid-treated cells the number of TNTs per image (frame) was calculated. For statistical analysis the data from 3 independent experiments were collected.

### Statistical analysis

The data are expressed as the mean ± standard deviation (s.d.) or the mean ± standard error of the mean (s.e.m.). For statistical analyses, the data were replicated in at least three experiments. Statistical significance between two groups was calculated using either a two-tailed Student’s t-test (TNT quantification after AA treatment) or a two-way repeated measures analysis of variance (ANOVA) followed by Dunnet’s test (treatment with cytoskeletal drugs, LD quantification). Differences were considered significant at p < 0.05 (*), p < 0.01 (**) or p < 0.001 (***).

## Additional Information

**How to cite this article**: Astanina, K. *et al.* Lipid droplets as a novel cargo of tunnelling nanotubes in endothelial cells. *Sci. Rep.* **5**, 11453; doi: 10.1038/srep11453 (2015).

## Supplementary Material

Supplementary Information

Supplementary Video S1

Supplementary Video S2

Supplementary Video S3
