# Supplementary material for: Lipid droplets as a novel cargo of tunnelling nanotubes in endothelial cells
Source: Sci Rep. 2015 Jun 22;5:11453. doi: 10.1038/srep11453 (PMC4476149; doi:10.1038/srep11453)
Supplement: Supplementary Information [file srep11453-s1.pdf]

Supplementary Information

Lipid droplets as a novel cargo of tunnelling nanotubes in endothelial cells

[1]Ksenia Astanina*, [2]Marcus Koch, [3]Christian Jüngst, [3]Andreas Zumbusch, [1]Alexandra K. Kiemer

[1]Department of Pharmacy, Pharmaceutical Biology, Saarland University, Saarbrücken, Germany, [2]Leibniz Institute for New Materials, Saarbrücken, Germany, [3]Department of Chemistry, Konstanz University, Konstanz, Germany

**Corresponding author. E-mail address:* k.astanina@mx.uni-saarland.de

Video S1

TNTs between cells are under tension. Once the anchor point (located on the substrate or on another cell) in the middle of the TNT is detached, the TNT straightens up. Scale bar, 20 μm. Time is indicated as h:min:s:ms. For still images see Fig. 2a.

Video S2

TNTs are often formed and maintained during mitosis. The transport of optically dense granules, or nodules, is observed inside TNTs (depicted with arrows). Scale bar, 20 μm. Time is indicated as h:min:s:ms. For still images see Fig. 2b.

Video S3

Transport of optically dense granules between cells in TNTs. Scale bar, 20 μm. Time is indicated as h:min:s:ms. For still images see Fig. 2c.

Figure S1

Cytotoxicity of cytoskeletal drugs (a) and fatty acids (b) was tested by MTT assay. (a) HMEC-1 were incubated with 200 nM jasplakinolide, 200 nM cytochalasin D, 100 nM taxol, or 100 nM nocodazole for 2 h. Control cells were treated with DMSO at the highest concentration used. (b)

HMEC-1 were incubated with either 80 µM arachidonic acid or 80 µM stearic acid for 24 h. Control cells were treated with ethanol at the highest used concentration. (a, b) The viability of control cells was set as 100%. The diagrams show the results of three independent experiments per treatment, with eight replicates each. Error bars denote s.d.. No significant differences between treated and control cells were found (Student's t-test).

Figure S2

HMEC-1 were stained either with CellTracker® Blue, or CellTracker® Orange. After the staining, the cells were mixed and seeded on coverslips. The images were taken 24 hours after seeding. TNTs are depicted with arrows. Scale bar, 20 µm.

Figure S3

Lipid droplets in HUVECs cells were stained with Nile red. Brightfield microscopy revealed the presence of TNTs in cultured HUVECs, Nile red staining showed that lipid droplets are present in the TNTs. Scale bar, 20 µm.

Figure S4

The presence of LDs in TNTs was demonstrated by CARS. Brightfield image (BF) of HMEC-1 cells has been recorded, followed by CARS. In order to avoid artifacts in the CARS microscopy due to absorption of LD540, the sample was stained and washed after recording of the CARS image and stained thereafter with LD540. The overlays of the channels are shown. Lipid droplets are depicted with arrows. Scale bar, 20 µm.

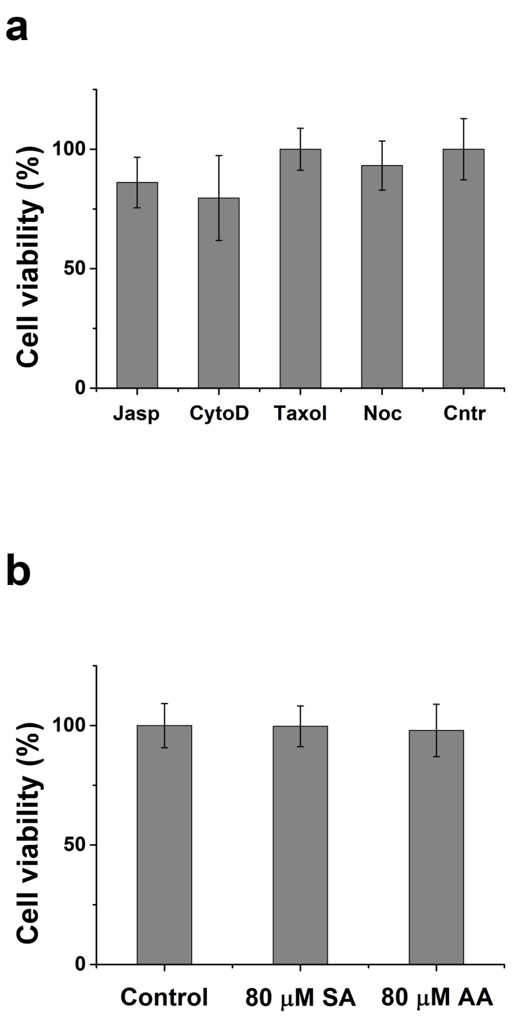

Supplementary figure S1

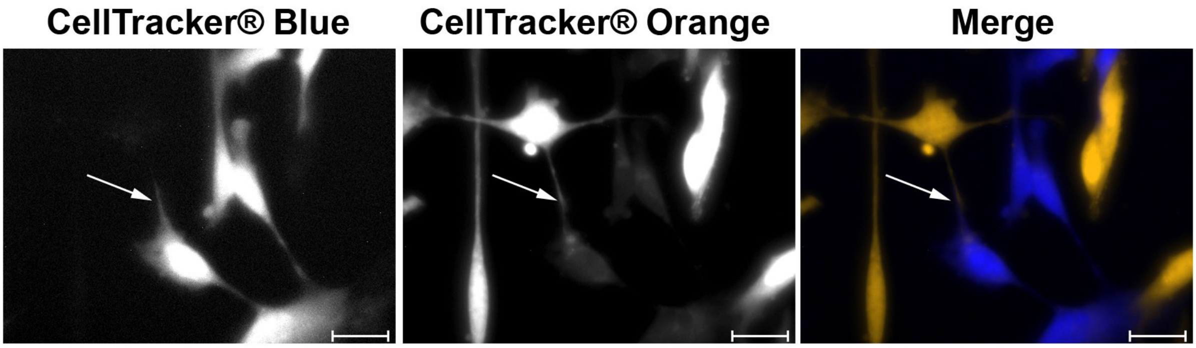

Supplementary figure S2

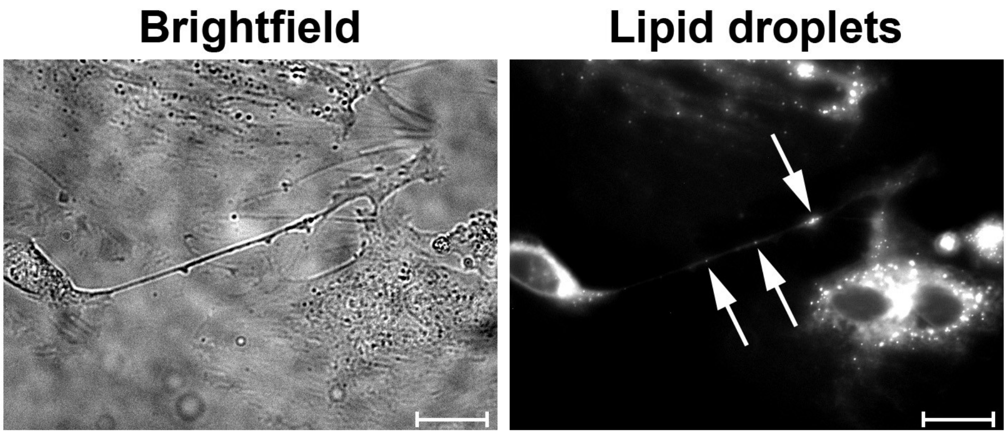

Supplementary figure S3

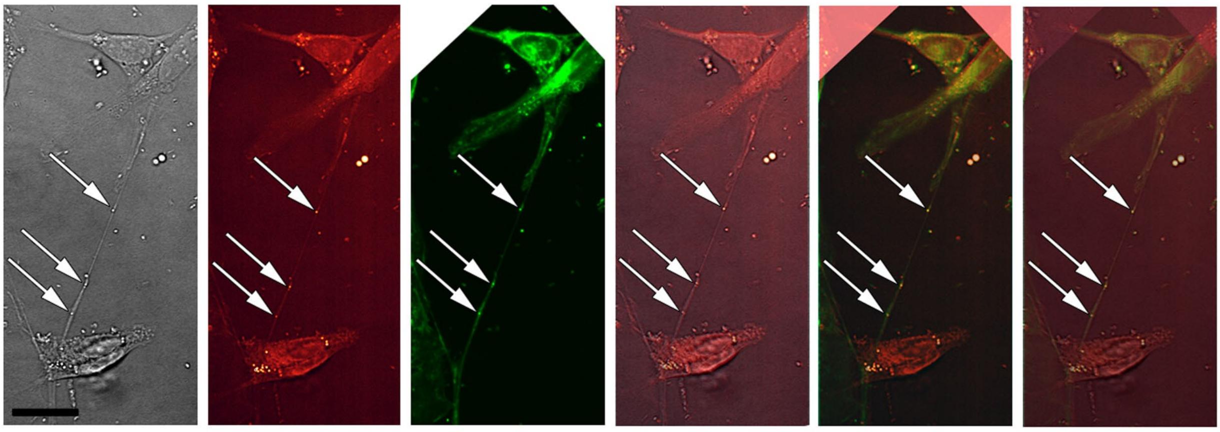

Supplementary figure S4
